# Anxiety in Patients With Acute Ischemic Stroke: Risk Factors and Effects on Functional Status

**DOI:** 10.3389/fpsyt.2019.00257

**Published:** 2019-04-17

**Authors:** Wei Li, Wei-Min Xiao, Yang-Kun Chen, Jian-Feng Qu, Yong-Lin Liu, Xue-Wen Fang, Han-Yu Weng, Gen-Pei Luo

**Affiliations:** ^1^Department of Neurology, Dongguan People’s Hospital, Dongguan, China; ^2^Department of Radiology, Dongguan People’s Hospital, Dongguan, China

**Keywords:** anxiety, depression, functional status, quality of life, stroke, cerebral hemispheric white matter

## Abstract

**Background:** Anxiety is prevalent after a stroke. The pathophysiological mechanisms underlying the development of poststroke anxiety (PSA) remain unclear. The aim of this study was to investigate the clinical and neuroimaging risk factors for development of PSA and examine the effects of PSA on activities of daily living (ADL) and quality of life (QOL) in Chinese patients with ischemic stroke.

**Methods:** Two hundred nineteen patients with acute ischemic stroke were recruited to the study. A series of comprehensive assessments, including Hamilton Anxiety Rating Scale (HARS), Hamilton Depression Rating Scale (HDRS), Lawton ADL Scale, and the Stroke-Specific Quality of Life (SSQOL) Scale, were conducted in the acute stage and 3 months after stroke. Magnetic resonance imaging assessment focused on evaluation of infarctions, white matter lesions, and brain atrophy.

**Results:** In the acute stage and 3 months after stroke, 34 (16%) and 33 (15%) patients had PSA, respectively. Multiple logistic regression analysis indicated that HDRS (OR = 1.269, 95% CI = 1.182–1.364, *P* < 0.001) and acute infarcts in cerebral hemispheric white matter (CHWM; OR = 2.902, 95% CI = 1.052–8.007, *P* = 0.040) were significant correlates of PSA in the acute stage of stroke. Three months after stroke, these correlates remained significant predictors, along with male sex. Multiple linear regressions showed that age, NIHSS, HARS, and HDRS in the acute stage were significant predictors for both ADL and SSQOL at 3 months after stroke.

**Conclusion:** Depressive symptoms are the major correlates of PSA while more severe PSA is associated with poorer ADL and health-related QOL. Acute lesions involving CHWM may correlate with PSA in ischemic stroke patients with mild-to-moderate neurologic deficits, supporting a lesion-location hypothesis in PSA.

## Introduction

Anxiety is prevalent after stroke and occurs in about one-quarter of stroke survivors (1, 2). Poststroke anxiety (PSA) may have a negative impact on quality of life (QOL) of stroke survivors, affecting their rehabilitation (3). Furthermore, one prospective study found that severe anxiety symptoms were associated with increased risk for incident stroke, independent of other risk factors (4). Despite high prevalence of anxiety after stroke, understanding of PSA is limited.

Risk factors related to PSA include depression (5–7), cognitive impairment (5, 8), fatigue (9), age (10–12), female sex (10, 12), lesion location (13, 14), and sleep disturbance (9, 15), indicating PSA might be multifactorial. Apart from stress due to acute ischemic stroke, the biological mechanisms of PSA should also be considered. Anxiety-related neural circuits span a wide range of brain structures, including subcortical white matter and the limbic system (16, 17). Neuroimaging techniques, e.g., magnetic resonance imaging (MRI), can locate the infarction precisely. Thus, studying the neuroimaging correlates of PSA may be helpful in understanding the pathophysiology of PSA. However, few studies have evaluated the association between PSA and neuroimaging variables. The lesion-location hypothesis of PSA might be presumed as the infarction may damage brain structures involved in anxiety. Recently, a large-scale MRI study involving 239 stroke patients was performed, but no association was found between brain lesion location and PSA (18). Thus, the underlying pathophysiological mechanisms of development of PSA remain unclear.

Functional status, including the ability to perform activities of daily living (ADL) and QOL, is an important outcome of stroke in many studies (3, 19, 20). However, few studies have explored the subsequent effect of PSA in the acute phase on patient QOL and functional outcomes in the chronic stage.

The purpose of this prospective study was twofold. The first purpose was to investigate the associated clinical and MRI risk factors for PSA, testing the lesion-location hypothesis of PSA. The second purpose was to examine the effects of PSA on patient ADL and QOL following ischemic stroke. We assumed that lesions in specific locations (e.g., structures related to emotional modulation) might be more likely to result in PSA. Severity of PSA in the acute stage is a significant factor independently contributing to poor ADL and QOL in the chronic stage.

## Methods

### Participants and Setting

Patients with first-ever or recurrent acute ischemic stroke admitted to the Department of Neurology, Dongguan People Hospital, between July 2013 and June 2014 were screened for this study. Patients were enrolled in the study if they met the following criteria: 1) age 40 to 80 years; 2) had an acute, first, or recurrent ischemic stroke that occurred within 7 days prior to admission; if they had a previous stroke, the modified Rankin Scale score before the index stroke was <2. Patients were excluded if they 1) had significant neurological illness other than stroke, e.g., Parkinson’s disease, brain tumor, or multiple sclerosis; 2) had no MRI scans or poor-quality MRI scans on admission; 3) had a severe stroke, which received a National Institutes of Health Stroke Scale (NIHSS) total score of ≥15; 4) had severe aphasia (defined as NIHSS best language subscore ≥2) or dysarthria; 5) had severe cognitive impairment, defined by a Mini-Mental State Examination (MMSE) total score of <17; 6) had a history of anxiety disorders, depression, substance abuse/dependence, or other psychiatric disorders before the index stroke; and 7) had comorbid severe diseases of the heart, lung, kidney, liver, or malignant tumors. This study was carried out in accordance with the recommendations of the World Medical Association’s Declaration of Helsinki. The study protocol was approved by the Ethics Committee of Dongguan People’s Hospital. Consent forms were obtained from the patients or their legally authorized representative.

### Collection of Demographic and Clinical Data

Patient demographics (age, sex, and education level) and clinical characteristics including vascular risk factors (e.g., hypertension, diabetes mellitus, hyperlipidemia, and smoking history) and previous stroke history were collected from medical records. The severity of stroke was assessed by the NIHSS from medical records.

### Assessment of PSA

The Chinese version of the 14-item Hamilton Anxiety Rating Scale (HARS) (21, 22) was used to evaluate anxiety symptoms in all participants in the acute stage when they were medically stable (5–14 days after the index stroke) and at the 3-month follow-up. Assessments of clinical anxiety were performed by two trained neurologists (WL and HW) who were blinded to the MRI results of the stroke survivors. The Chinese version of the 14-item HARS has been widely used in the Chinese population, as well as in Chinese stroke patients (22), indicating good reliability and validity. PSA in this study was defined by a HARS score ≥14 (22). HARS was repeatedly conducted at 3 months after stroke by the same raters. PSA was defined as a HARS score ≥14 at 3 months after stroke. If the patients were diagnosed with anxiety at baseline and received anti-anxiety treatment, they were also judged to have PSA even if they had a HARS score lower than 14.

### Assessment of Other Psychological Status in the Acute Stage of Stroke

The Chinese version of the MMSE (scores range from 0 to 30, with lower scores indicating greater deficits) (23) was used to measure basic cognitive function by the two trained neurologists (WL and HW). They also administered the Chinese version of the 24-item Hamilton Depression Rating Scale (HDRS) (22, 24), which was used to evaluate the severity of depressive symptoms, with an internal consistent Cronbach’s α = 0.88–0.99 (22).

### Assessment of Functional Status at 3 Months After Stroke

The two raters administered the Lawton ADL Scale (25) and the Chinese version of the Stroke-Specific Quality of Life (SSQOL) Scale (26). The Lawton ADL Scale, which contains six items assessing self-maintenance and eight items evaluating instrumental activities, was used to measure functional level of patients with stroke. Each item was rated from 1 to 4, and the total ADL score was calculated by summing the scores of all items. Higher scores indicate poorer performance. The test–retest kappa of the Chinese version of the Lawton ADL Scale is 0.502 (22). The Chinese version of the SSQOL Scale, which was used to assess patient QOL and proved to have good reliability and validity, consists of 49 questions grouped into 12 domains, with scores rated from 1 (worst outcome) to 5 (best outcome). The internal consistent reliability is high (Cronbach’s α = 0.76) (26, 27).

Before the first interview, the two neurologists selected 10 patients with ischemic stroke to test the interrater reliability of the rating instruments. The intraclass correlation coefficients (ICCs) of the above scales between the two raters ranged between 0.83 and 0.91.

### Magnetic Resonance Imaging Assessment

MRI acquisition was performed using a 1.5-T scanner (Achieva Phillip Medical System, Best, the Netherlands) within 7 days of the index stroke. The sequences of MRI scanning included diffusion-weighted imaging (DWI), gradient echo sequences, and T1- and T2-weighted, fluid-attenuated inversion recovery sequences. A trained neurologist (YL), who was blinded to patient clinical information, assessed the MRI variables as follows:

1. Infarcts: The location, number, and volume of acute infarcts were examined in DWI. The sites of acute infarcts were denoted by brain region as follows: frontal, parietal, temporal, and occipital lobes; corpus callosum; coronal radiate; centrums semiovale; internal capsule; basal ganglia; thalamus; brainstem; and cerebellum. If the patient had infarcts in more than two sites, both sites would be recorded as presence. Cerebral hemispheric white matter (CHWM) was defined as any supratentorial white matter structure including the corpus callosum, coronal radiate, centrums semiovale, and internal capsule.
2. The total volume was calculated by multiplying the total area by the sum of the slice thickness and the gap. The number of old lacunar infarcts was also recorded.
3. White matter lesions (WMLs). The extent of WMLs was graded using the four-point scale of Fazekas et al. (28). Deep white matter hyperintensities (DWMH) and periventricular hyperintensities (PVH) were scored on fluid-attenuated inversion recovery (FLAIR) images.
4. Ventricle-to-brain ratio (VBR). VBR is an indicator of global brain atrophy (29). The slice showing the longest vertical length of the lateral ventricle at the middle was selected. The VBR was defined as the ratio of the diameter of the width of the lateral ventricle divided by the width of the brain along the same line (30).
5. Medial temporal lobe atrophy (MTLA). MTLA was measured using Schelten’s scale (31). This visual rating scale yields standard images with different severity of MTL atrophy on coronal MRI sections, ranging from 0 to 4, from “no atrophy” to “severe atrophy.” The MTLA score was determined using the sum of left and right medial temporal lobes.

Intrarater reliability activities were performed on 10 patients by the same MRI rater at two time points (interval ≥2 months). The intrarater agreements of the MRI measurements were good to excellent, as reported in our previous study (32).

### Statistical Analysis

All statistical tests were performed using SPSS for Windows (Release 16.0, SPSS Inc., Chicago, IL, USA). In the acute stage of stroke, all patients were divided into two groups, the PSA and non-PSA groups, according to the HARS cutoff. The demographic and clinical variables were compared between the PSA and non-PSA groups using χ^2^ test, two independent *t* tests, or Mann–Whitney *U* tests, as appropriate, in order to screen for potential predictors. Variables with *P* < 0.1 in univariate comparisons were entered as independent variables in multiple stepwise logistic regression analysis with PSA as the dependent variable. The same statistical procedures were performed at 3 months after stroke. Subsequently, multiple linear regressions were performed to explore the effects of HARS in the acute stage on ADL and SSQOL at 3 months after stroke (ADL and SSQOL were used as dependent variables) after adjusting for age, sex, NIHSS, and HDRS. The significance level was set at 0.05 (two-sided).

## Results

A total of 435 patients aged 40 to 80 years with acute ischemic stroke were admitted and screened. Two hundred nineteen patients (50.3%) fulfilled the study criteria and were included in the study. Compared to those who were excluded, participating patients were younger (61.4 ± 11.2 *vs.* 64.8 ± 12.7 years; *P* < 0.001), had a lower NIHSS score at admission (median, 3.0 [range, 0–15] *vs*. 5.0 (0–35), *P* < 0.001), but had a comparable frequency of male sex (73.1% *vs*. 68.1%; *P* = 0.252).

### Demographic and Clinical Characteristics

The study cohort consisted of 219 patients who satisfied the study criteria (**Table 1**). One patient died and three patients were lost to follow-up before the 3-month assessment. In the acute stage and 3 months after the index stroke, there were 34 (15.5%) and 33 (15.1%) patients who were judged to have PSA, respectively. Compared to patients without PSA, patients with PSA were more likely to be female and to have more severe depressive symptoms (**Table 1**). No MRI variables were significantly different between the two groups, although patients with PSA trended toward more CHWM infarcts in both the acute stage and 3 months after stroke (*P* = 0.075 and *P* = 0.071, respectively; **Table 2**).

**Table 1:** Comparisons of demographic and clinical variables between the PSA and non-PSA groups.

Variables	PSA in the acute stage		*P*	PSA at 3 months after stroke		*P*
	Yes (*n* = 34)	No (*n* = 185)		Yes (*n* = 33)	No (*n* = 182)	
Age (years)*	63.1 (9.6)	60.9 (11.6)	0.281	63.8 (9.7)	60.7 (11.7)	0.141
Female sex (*n*, %)^†^	14 (41.2%)	45 (24.3%)	0.042	14 (42.4%)	43 (23.6%)	0.024
Education level^†^						
Secondary and tertiary	10 (29.4%)	60 (32.4%)	0.728	8 (24.2%)	62 (34.1%)	0.268
Hypertension (*n*, %)^†^	24 (70.6%)	139 (75.1%)	0.576	24 (72.7%)	135 (74.2%)	0.862
Diabetes (*n*, %)^†^	9 (26.5%)	49 (26.5%)	0.998	8 (24.2%)	50 (27.5%)	0.700
Hyperlipidemia (*n*, %)^†^	12 (37.5%)	62 (36.7%)	0.930	8 (26.7%)	65 (38.9%)	0.201
Previous stroke (*n*, %)^†^	6 (17.6%)	27 (14.6%)	0.647	6 (18.2%)	26 (14.3%)	0.563
NIHSS on admission^‡^	4 (3–5)	3 (2–5)	0.101	4 (2–7)	3 (2–5)	0.069
MMSE^†^	24.1 (3.9)	25.3 (4.8)	0.175	23.7 (4.0)	25.4 (4.8)	0.055.
HDRS^‡^	19.6 (6.2)	6.2 (5.5)	<0.001	18.9 (9.2)	3.9 (3.7)	<0.001
HARS^‡^	18.2 (4.9)	4.8 (3.6)	<0.001	16.4 (6.8)	5.2 (4.3)	<0.001

PSA, poststroke anxiety; NIHSS, National Institutes of Health Stroke Scale; MMSE, Mini-Mental State Examination; HDRS, Hamilton Depression Rating Scale; HARS, Hamilton Anxiety Rating Scale.

*Mean (SD), t test; ^†^n (%), chi-square test; ^‡^ median (25%Q–75%Q), Mann–Whitney U test.

**Table 2:** Comparisons of MRI variables between the PSA and non-PSA groups.

Variables	PSA in the acute stage		*P*	PSA at 3 months after stroke		*P*
	Yes (*n* = 34)	No (*n* = 185)		Yes (*n* = 33)	No (*n* = 182)	
Acute infarcts (*n*, %)*						
Frontal lobe	4 (11.8%)	30 (16.2%)	0.615	4 (12.1%)	29 (15.9%)	0.793
Parietal lobe	3 (8.8%)	21 (11.4%)	1.000	4 (12.1%)	19 (10.4%)	0.761
Temporal lobe	1 (2.9%)	14 (7.6%)	0.476	1 (3.0%)	14 (7.7%)	0.477
Occipital lobe	1 (2.9%)	12 (6.5%)	0.697	1 (3.0%)	12 (6.6%)	0.697
Corpus callosum	2 (5.9%)	8 (4.3%)	0.656	2 (6.1%)	8 (4.4%)	0.653
Corona radiata	12 (35.3%)	46 (24.9%)	0.205	13 (39.4%)	46 (25.3%)	0.094
Centrums semiovale	2 (5.9%)	18 (9.7%)	0.746	3 (9.1%)	17 (9.3%)	1.000
Internal capsule	6 (17.6%)	18 (9.7%)	0.174	5 (15.2%)	19 (10.4%)	0.429
Basal ganglia	9 (26.5%)	31 (16.8%)	0.178	9 (27.3%)	31 (17.0%)	0.164
Thalami	2 (5.9%)	16 (8.6%)	0.746	1 (3.0%)	16 (8.8%)	0.481
Brainstem	4 (11.8%)	38 (20.5%)	0.343	5 (15.2%)	36 (19.8%)	0.533
Cerebellum	0 (0.0%)	12 (6.5%)	0.221	0 (0.0%)	12 (6.6%)	0.220
CHWM	19 (55.9%)	73 (39.5%)	0.075	19 (57.6%)	74 (40.7%)	0.071
Volume of acute infarcts (ml)^†^	0.9 (0.5–3.8)	0.9 (0.3–3.0)	0.753	1.0 (0.4–4.0)	0.9 (0.2–2.9)	0.578
Number of acute infarcts^†^	1 (1–2)	1 (1–2)	0.578	1 (1–2)	1 (1–2)	0.594
Number of old infarcts^†^	0 (0–2)	0 (0–2)	0.412	0 (0–2)	0 (0–2)	0.442
PVH	1 (0–2)	1 (1–2)	0.298	1 (0–2)	1 (1–2)	0.879
DWMH	1 (0–2)	1 (1–2)	0.330	1 (0–2)	1 (1–2)	0.805
VBR^‡^	17.9 (5.4)	18.8 (4.2)	0.248	18.4 (5.8)	18.8 (4.2)	0.689
MTLA^†^	0 (0–2.25)	0 (0–2)	0.941	0 (0–3)	0 (0–2)	0.989

PSA, poststroke anxiety; CHWM, cerebral hemispheric white matter; PVH, periventricular hyperintensities; DWMH, deep white matter hyperintensities; VBR, ventricle-to-brain ratio; MTLA, medial temporal lobe atrophy.

*n (%), chi-square test; ^†^median (25%Q–75%Q), Mann–Whitney U test; ^‡^mean (SD), t test.

### Correlates of Poststroke Anxiety in the Acute Stage of Stroke

HDRS, sex, and acute infarcts in CHWM were evaluated by multiple logistic regressions. HDRS (odds ratio [OR] = 1.269, 95% CI = 1.182–1.364, *P* < 0.001) and acute infarcts in CHWM (OR = 2.902, 95% CI = 1.052–8.007, *P* = 0.040) were significant correlates of PSA in the acute stage of stroke (**Table 3**).

**Table 3:** Correlates of PSA in the acute stage of ischemic stroke.

Variables	β	*P*	OR	95% CI
HDRS	0.238	<0.001	1.269	1.182–1.364
Acute infarcts in CHWM	1.065	0.040	2.902	1.052–8.007
Sex (female)	2.521	0.112	2.307	0.811–6.564-

Total R^2^ = 0.536; PSA, poststroke anxiety; HDRS, Hamilton Depression Rating Scale; CHWM, cerebral hemispheric white matter.

### Correlates of Poststroke Anxiety at 3 Months After Stroke

HDRS, sex, NIHSS, MMSE, and acute infarcts in CHWM were evaluated by multiple logistic regression. HDRS (OR = 1.232, 95% CI = 1.150–1.320, *P* < 0.001), female sex (OR = 3.214, 95% CI = 1.124–9.189, *P* = 0.029), and acute infarcts in CHWM (OR = 2.904, 95% CI = 1.033–8.162, *P* = 0.043) significantly correlated with PSA (**Table 4**).

**Table 4:** Correlates of PSA at 3 months after stroke.

Variables	β	*P*	OR	95% CI
HDRS	0.209	<0.001	1.232	1.150–1.320
Acute infarcts in CHWM	1.066	0.043	2.904	1.033–8.162
Sex (female)	1.168	0.029	3.214	1.124–9.189
MMSE	1.524	0.217	0.931	0.831–1.044
NIHSS	0.173	0.678	0.942	0.801–1.108

Total R^2^ = 0.491; PSA, poststroke anxiety; HDRS, Hamilton Depression Rating Scale; MMSE, Mini-Mental State Examination; CHWM, cerebral hemispheric white matter.

### Effects of Anxiety in the Acute Stage on Activities of Daily Living and Stroke-Specific Quality of Life at 3 Months After Stroke

Multiple linear regressions showed that age, NIHSS, HARS, and HDRS in the acute stage were significant predictors for both ADL and SSQOL at 3 months after stroke (**Table 5**). Patients with PSA in the acute stage were more likely to have a poorer performance in ADL and SSQOL at 3 months after stroke.

**Table 5:** The effects of PSA in the acute stage on ADL and SSQOL at 3 months after stroke.

Dependent variable	Independent variables			
	3-month ADL		3-month SSQOL	
	Adjusted β	*P*	Adjusted β	*P*
HARS in the acute stage	0.282	<0.001	−0.252	0.014
Age	0.155	0.011	−0.142	0.012
Sex (female)	−0.013	0.837	0.087	0.124
NIHSS	0.318	<0.001	−0.225	0.001
HDRS in the acute stage	0.281	<0.001	−0.258	0.014
*R* ^2^	0.255		0.360	

PSA, poststroke anxiety; ADL, activities of daily living; SSQOL, Stroke-Specific Quality of Life; HDRS, Hamilton Depression Rating Scale; HARS, Hamilton Anxiety Rating Scale; NIHSS, National Institutes of Health Stroke Scale.

### Sensitivity Analysis After Excluding Patients With Previous Stroke

Analyses including only patients with their first-ever stroke are summarized in the supplemental tables. Acute infarcts in CHWM remained a significant correlate of PSA in the acute stage, but not at 3 months after stroke. HARS score in the acute stage significantly contributed to poorer ADL and SSQOL 3 months after stroke after adjusting for age, sex, NIHSS, and HDRS.

## Discussion

In this prospective and longitudinal study, we found that frequency of PSA in the acute stage and 3 months after a mild-to-moderate ischemic stroke was 15.5% and 15.1%, respectively. HDRS and acute infarcts in CHWM correlated with PSA in both the acute stage and 3 months after stroke. Severity of PSA was a significant indicator for both ADL and SSQOL. To the best of our knowledge, studies investigating the effects of PSA on functional status are very limited. Our study represents a significant contribution to literature on the significance of PSA.

Anxiety symptoms were common after stroke. A meta-analysis study estimated that PSA affected 25% of stroke survivors (1). A summary of studies on PSA is shown in **Table 6**. In our study, we used HARS to assess anxiety symptoms with a cutoff of mean HARS ≥14 and found that frequency of PSA was about 15% in stroke survivors, which was lower than most previous studies (33–37). This may be due to the inclusion of a stroke sample with relatively mild neurological deficits (median NIHSS, 4) and exclusion of severe neurologic deficits or aphasia. Patients excluded from this study might be more likely to have PSA. Differences in assessment tools for PSA might also contribute to the differences between our results and previous studies.

**Table 6:** Summary of studies assessing the correlates or prevalence of anxiety in patients with ischemic stroke.

Study	Source of sample and sample size	Mean age	Sex (male)	Assessment tool of PSA	Assessment of ADL	Assessment of SSQOL	MRI assessment	Assessment time point	Frequency of PSA	Significant correlates
The present study	Hospital (*n* = 219)	61.4	73.1%	HARS	Yes	Yes	Yes	Acute stage and 3 m	15.5% (acute) 15.1% (3 m)	HDRS, acute infarcts in CHWM (acute) HDRS, male, acute infarcts in CHWM (3 m)
Schultz et al. (10)	Hospital (*n* = 142)	58.1	57%	*DSM-IV*	Yes	No	No	3 m 6 m 1 year 2 years	19% (acute) 22.1% (3 m) 25.3% (6 m) 11.4% (1 year) 18.2% (2 years)	Depression, ADL, and female
Chun et al. (38)	Hospital (*n* = 175)	69.6	60%	*DSM-IV*	No	Yes	No	3 m	22%	Pre-stroke depression, pre-stroke anxiety, EQ-5D5L, and young age
De Wit et al. (33)	European rehabilitation centers (*n* = 532)	69.5	53.3%	HADS-A	No	No	No	2 m 4 m 6 m	25% (2 m) 22% (4 m) 22% (6 m)	No significant correlates
Vuletić et al. (34)	Hospital (*n* = 40)	71.1	50%	HADS-A	No	No	No	Acute stage	40%	MMSE, BI
Wu et al. (36)	Hospital (*n* = 226)	63.13	62.84%	HARS	No	No	No	1 m	26.6%	Vitamin D deficiency
D’Aniello et al. (35)	Hospital (*n* = 81)	62	59.2%	HADS-A	No	No	No	Undefined	55.6%	No correlates
Vicentini et al. (12)	Hospital (*n* = 34)	NR	64.7%	BAI	No	No	Yes	Undefined	11.8%	Disruption of DMN
Liu et al. (18)	Hospital (*n* = 203)	63.5	64.5%	HARS	No	No	Yes	Acute stage	24.1%	MDA, GPX, SOD, and CAT
Lincoln et al. (37)	European rehabilitation centers (*n* = 220)	67.5	54%	HADS-A	No	No	No	5 years	29%	No correlates
Broomfield et al. (39)	Community (*n* = 3831)	70.39	55.3%	HADS-A	No	No	No	Undefined	16.1%	Age, gender, and socioeconomic deprivation
Tang et al. (13)	Hospital (*n* = 693)	65.6	61%	HADS-A	No	No	Yes	3 m	6.1%	Frontal infarcts

PSA, poststroke anxiety; ADL, activities of daily living; SSQOL, Stroke-Specific Quality of Life; HARS, Hamilton Anxiety Rating Scale; HDRS, Hamilton Depression Rating Scale; CHWM, cerebral hemispheric white matter; DSM-IV, Diagnostic and Statistical Manual of Mental Disorders-IV-Text Revision; EQ-5D5L, EuroQoL-5D5L; HADS-A, Hospital Anxiety and Depression Scale–Anxiety; MMSE, Mini-Mental State Examination; BI, Barthel Activities of Daily Living Index; BAI, Beck Anxiety Inventory; DMN, Default Mode Network; MDA, malondialdehyde; GPX, glutathione peroxidase; SOD, superoxide dismutase; CAT, catalase; NR, not recorded.

Sudden occurrence of neurological deficits might cause stress or anxiety in stroke patients. We assessed PSA at two time points, including the acute stage (5–14 days) and months after stroke. The time point of 5–14 days was chosen for the first time point because this is when patients are typically medically stable. The 3-month time point was selected as the acute effects of stress related to an adverse life event might have diminished, and is a common time point used in previous studies (5, 13). As PSA might be multifactorial, we collected comprehensive data to the extent possible, including clinical, physical, psychological, and neuroimaging variables. The present study showed that PSD was significantly associated with PSA both in the acute stage and 3 months after stroke, indicating that PSD and PSA may share a common pathophysiological mechanism. This comorbidity has been confirmed by other studies (2, 5, 37).

Available clinical data on the relationship between PSA and lesion location are conflicting. Tang et al. (13) found that patients with acute frontal lobe infarction were more likely to have PSA. Similar to other studies (8, 18, 40), we could not locate a single lesion location that was directly related to PSA. However, we found that patients with acute infarction in the CHWM were more likely to have PSA in the acute stage of stroke, as well as 3 months after stroke. This finding has not been reported previously. CHWM includes a wide range of regions of connected neural fibers in the cerebral hemisphere, e.g., corpus callosum, corona radiata, centrums semiovale, and internal capsule. Neural circuits associated with emotion regulation are widely distributed in the cerebral hemispheres, such as the fronto-subcortical circuits or the limbic system (41, 42). Brain white matter abnormalities have also been implicated in development of anxiety (17). Thus, acute CHWM lesions are logical potential contributors to PSA. However, the wide range of OR in CHWM in prediction of PSA indicates that this preliminary finding should be carefully repeated in further studies. Furthermore, after excluding patients with previous strokes, CHWM only contributed to PSA in the acute stage, but not 3 months after stroke.

The role of CHWM in development of PSA remains unclear. Recently, studies have focused on lesions involving neuronal network or circuits rather than single locations. Fornito et al. (43) reported that functional neuronal network disruption may be more critical than lesion location to explain PSA. Vicentini et al. (12) reported that PSA was not associated with infarct location but correlated with disruption of the default mode network (DMN) in the brain. Accordingly, examining the effects of the integrity of brain networks or neural circuits rather than a single location on PSA might be another direction for further research.

The severity of PSA in the acute stage was inversely associated with performance of ADL in this study. Schultz et al. (10) reported that association of anxiety and impairment in ADL were present only at the initial evaluation (in the acute stage of ischemic stroke), with independent effects only for women. It can be postulated that PSA patients may have poor adherence to rehabilitative efforts because of a significant decrease in both physical and mental energy, which, in turn, impairs performance of ADL.

Stroke frequently reduces the level of health-related QOL (HRQOL) of survivors. Our study indicated that the severity of PSA in the acute stage was a significant contributor to poorer SSQOL 3 months after stroke. PSA may reduce physical and mental energy, motivation, and activity, which then inversely affects HRQOL. A cross-sectional study also found that poorer QOL was associated with greater levels of physical disability, anxiety, and depression, and reduced social interaction (44). Thus, assessment and intervention of PSA as well as PSD in the acute stage may be helpful to predict outcomes of functional status.

Our study has several strengths. First, we conducted a face-to-face interview to evaluate functional and psychological measures, which was rarely reported in other studies. Second, we obtained comprehensive MRI data from all participants. However, there were also several limitations to our study. First, only patients with mild-to-moderate ischemic stroke without severe cognitive impairment and aphasia were recruited, which limits the generalization of our findings. Second, we did not collect medication or rehabilitation after discharge, though most patients would have follow-up visits with neurologists or general physicians in community clinics. Third, we only used a screening tool (HDRS) rather than the standard psychiatric interview to define PSA, as there are no sufficient psychiatric professionals in our hospital. Lastly, the associations between PSA and QOL or ADL did not indicate causality due to the study design.

In general, anxiety is common in the acute and chronic stages of ischemic stroke with mild-to-moderate neurologic deficits. The lesion-location hypothesis of PSA might be relevant but remains uncertain. PSA in the acute stage may have a significant impact on ADL and HRQOL in stroke patients in the chronic stage. Early detection of anxiety symptoms may facilitate functional recovery and improve QOL in stroke patients. Careful evaluation of PSA should be integrated into clinical care of stroke patients.

## Ethics Statement

This study was carried out in accordance with the recommendations of “Operational Guidelines for Ethics Committees That Review Biomedical Research, World Health Organization (2000), Ethics Committee of Dongguan People’s Hospital” with written informed consent from all subjects. All subjects gave written informed consent in accordance with the Declaration of Helsinki. The protocol was approved by the “Ethics Committee of Dongguan People’s Hospital.”

## Author Contributions

YC and WX designed the study. WL, JQ, and GL screened and collected the patients. WL and HW performed the psychological assessments. XF designed and trained the MRI assessment. YL assessed the MRI variables. WL and YC wrote the manuscript.

## Funding

This study was funded by the Medical Scientific Research Foundation of Guangdong Province, China (Grant No: B2011349).

## Conflict of Interest Statement

The authors declare that the research was conducted in the absence of any commercial or financial relationships that could be construed as a potential conflict of interest.
